# Identifying Predictors of Nursing Home Admission by Using Electronic Health Records and Administrative Data: Scoping Review

**DOI:** 10.2196/42437

**Published:** 2023-11-20

**Authors:** Eunkyung Han, Hadi Kharrazi, Leiyu Shi

**Affiliations:** 1 Ho-Young Institute of Community Health, Paju, Republic of Korea; 2 Asia Pacific Center For Hospital Management and Leadership Research, Johns Hopkins Bloomberg School of Public Health, Baltimore, MD, United States; 3 Department of Health Policy and Management, Johns Hopkins School of Public Health, Baltimore, MD, United States; 4 Division of Biomedical Informatics and Data Science, Johns Hopkins School of Medicine, Baltimore, MD, United States

**Keywords:** prediction model, nursing home admission, electronic health record, EHR, administrative claims data, administrative data, claims data, health record, medical record, long-term care, nursing home, elder care, geriatric, gerontology, machine learning, PRISMA, scoping review, search strategy, aging, older adult

## Abstract

**Background:**

Among older adults, nursing home admissions (NHAs) are considered a significant adverse outcome and have been extensively studied. Although the volume and significance of electronic data sources are expanding, it is unclear what predictors of NHA have been systematically identified in the literature via electronic health records (EHRs) and administrative data.

**Objective:**

This study synthesizes findings of recent literature on identifying predictors of NHA that are collected from administrative data or EHRs.

**Methods:**

The PRISMA-ScR (Preferred Reporting Items for Systematic Reviews and Meta-Analyses extension for Scoping Reviews) guidelines were used for study selection. The PubMed and CINAHL databases were used to retrieve the studies. Articles published between January 1, 2012, and March 31, 2023, were included.

**Results:**

A total of 34 papers were selected for final inclusion in this review. In addition to NHA, all-cause mortality, hospitalization, and rehospitalization were frequently used as outcome measures. The most frequently used models for predicting NHAs were Cox proportional hazards models (studies: n=12, 35%), logistic regression models (studies: n=9, 26%), and a combination of both (studies: n=6, 18%). Several predictors were used in the NHA prediction models, which were further categorized into sociodemographic, caregiver support, health status, health use, and social service use factors. Only 5 (15%) studies used a validated frailty measure in their NHA prediction models.

**Conclusions:**

NHA prediction tools based on EHRs or administrative data may assist clinicians, patients, and policy makers in making informed decisions and allocating public health resources. More research is needed to assess the value of various predictors and data sources in predicting NHAs and validating NHA prediction models externally.

## Introduction

Aging is one of the most impactful medical and social demographic challenges across the globe [1]. By 2050, with the older population projected to reach 1.5 billion people, the proportion of older adults will be approximately 16% of the world’s total population [1,2]. In response, many countries have implemented long-term care within their health systems. Long-term care, which is defined as a variety of individualized, well-coordinated services that promote independence for people with functional limitations, is provided over an extended period in both community settings and institutional settings, including nursing homes [3].

Among older adults, nursing home admissions (NHAs) are a major driver of costs and are extensively studied along with other adverse outcomes, such as hospitalization and mortality [4,5]. Identifying the common predictors of NHA is considered a key factor in delaying the entry into long-term care services [2] and in enhancing the development and application of preadmission assessments for managing long-term care utilization among older adults [6]. Furthermore, identifying predictors of NHA among older adults can increase the understanding of modifiers for functional status, dependency, and utilization of health services for older patients [7,8]. As Gauler et al [9] reported, NHA can be considered as “less of an endpoint and instead as an important transition where associated factors operate to influence outcomes after admission” [9]. As choosing NHA is a crucial and challenging decision for both patients and caregivers, it may require additional tools to facilitate shared decision-making. Therefore, a model that can predict an individual’s risk of admission based on NHA predictors can help improve communication with patients and the implementation of clinical and policy preventive measures.

Clinical data sources, such as administrative data and electronic health record (EHR) technology, have become central in developing digital health tools throughout the health care spectrum [10]. Electronic data, including administrative claims and EHR data, are widely available across large populations, including older adults. These data sources can offer information across expanded follow-up periods, thereby improving the quality of study findings [11]. Predictors extracted from insurance claims or EHR data can be particularly useful for predicting NHA among older adults on a population scale, including the development of risk prediction models.

Administrative data are commonly leveraged to study health care delivery, benefits, harms, and costs [11]. Among these data, claims data have been increasingly used and are reported to be more structured and consistent, while EHR data have more in-depth information on each patient. Most notably, EHRs can contain a wide range of clinical data and narrative clinical notes that provide important information about a patient’s medical history, symptoms, and the clinical reasoning that underpins treatment decisions. Both data sources cover a different range of factors and are often complementary to each other [12].

It is unclear what NHA predictors have been systematically identified in the literature via EHRs and administrative claims data. To address this gap in knowledge, this study aimed to conduct a scoping review to map the recent evidence on assessing predictors of NHA among older adults. This review aimed to address the following research questions: (1) what were the predictors or associated factors of NHAs among past studies, and (2) what prediction models were constructed or validated to predict NHA?

## Methods

This scoping review was conducted per the Arksey and O’Malley framework [13] and the Joanna Briggs Institute methodology [14]. To report the study selection process, the extended PRISMA-ScR (Preferred Reporting Items for Systematic Reviews and Meta-Analyses extension for Scoping Reviews) guidelines were used [15].

### Identifying Relevant Studies

The electronic databases used in this review included the PubMed and CINAHL databases. Table S1 in Multimedia Appendix 1 provides additional information about the search terms used for this review.

### Search Strategies

#### Inclusion Criteria

As shown in Table 1, this review included studies that (1) were peer reviewed, (2) researched long-term care, (3) were authored in English, and (4) were deemed original research. Search terms were selected based on the following elements: population, concept, and context [16]. Each element was further refined, as follows: the population of interest was older adults at risk of NHA; the concept studied was the prediction of health, health care utilization, or health expenditure; and the studied context was the use of EHRs, electronic medical records, or administrative claims data. We limited the studies to those published after 2011, as the last review of similar studies already included publications published by the year 2011 [17]. More specifically, studies were limited to those published between January 1, 2012, and March 31, 2023.

**Table 1.** Eligibility criteria used in this review.

	Inclusion criteria	Exclusion criteria
Article type	Peer-reviewed studies deemed original research	Studies that were not peer reviewed, as well as reviews, abstracts, commentaries, editorials, letters to the editor, study protocols, and gray literature
Language	English	Languages other than English
Population characteristics	Older adults at risk of NHA^a^	Older adults who already reside in nursing homes, skilled nursing facilities(SNFs), or hospices
Predictor characteristics	At least 1 predictor related to NHA	No predictor reported related to NHA

a NHA: nursing home admission.

#### Exclusion Criteria

The following studies were excluded from this review: (1) studies with patients who were already residing in nursing homes and were thus missing NHA data; (2) studies that reported predictors of adverse outcomes among older adults but not predictors of NHA (eg, studies that only reported hospital or hospice admission); and (3) studies that only focused on SNFs, as our review focused on longer-term residence (typically longer than 1 y) rather than on short-term rehabilitation environments [2]. Reviews, abstracts, editorials, letters to the editor, and study protocols (with no outcome results) were also excluded (Table 1).

### Selection of Sources of Evidence, Data Charting, and Synthesis of Results

All authors participated in the planning of screening and data extraction, as well as in the construction of search strategies. One author (EH) evaluated the titles, abstracts, and full texts of all publications identified through systematic searches.

A data charting form was developed and refined, incorporating the final variables, such as study sample, country, the dependent variables used in the study, the independent variables used in the study, data source, statistical analysis, findings or conclusions, settings, and implications. Key study characteristics and detailed information were extracted from eligible studies and recorded, using the charting form. Furthermore, we categorized and synthesized the sources of evidence that contained explicit information on leveraging electronic data and addressing the practical implementation of the outcome of NHA risk prediction. The study selection and data charting processes were amended, with any disagreements among the authors being addressed and resolved consensually.

Based on the results obtained from data extraction and charting, we found that the identified studies covered varying health care settings and timings of nursing home utilization. Accordingly, we established distinct phases to differentiate the timings and settings of risk prediction (Figure S1).

## Results

### Selection of Sources of Evidence

**Figure 1.**
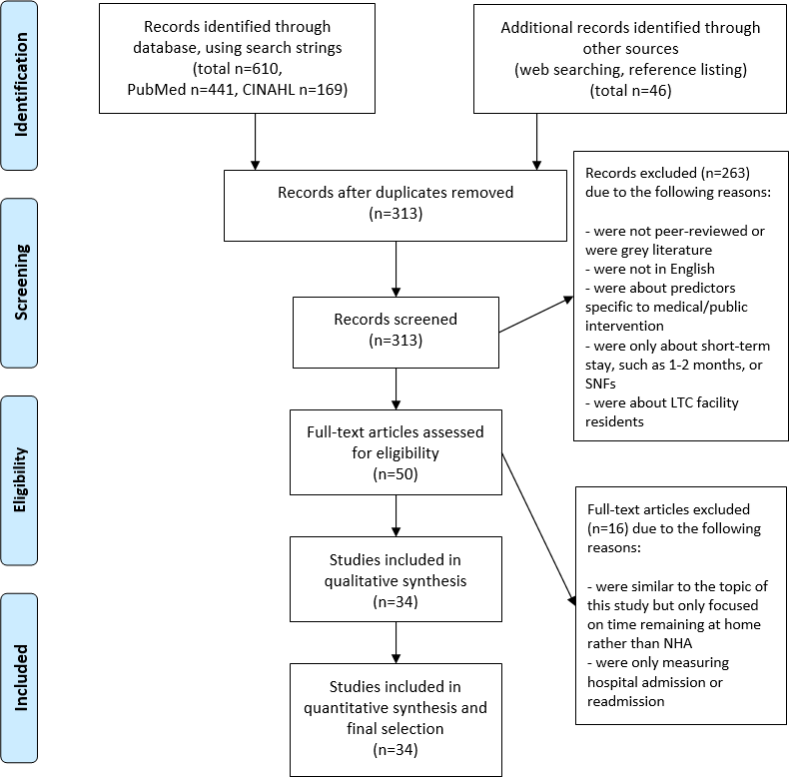
PRISMA (Preferred Reporting Items for Systematic Reviews and Meta-Analyses) flow diagram [51]. LTC: long-term care; NHA: nursing home admission; SNF: skilled nursing facility.

The initial search identified 610 articles across all search engines. Searching through the references of past reviews provided an additional 46 articles. A total of 313 articles remained after removing the duplicates. Screening the titles and abstracts of the 313 articles resulted in the exclusion of 263 articles. The full texts of the remaining 50 publications were reviewed, which resulted in the exclusion of 16 articles. A total of 34 peer-reviewed original research publications were selected for final inclusion in this review (Figure 1). Tables S1-S4 in Multimedia Appendix 1 [8,18-50] present the search strings and the comprehensive characteristics of the sources of evidence included in this study.

### Results of Individual Sources of Evidence

#### Patient Population

Different patient populations with various (typically chronic) conditions (eg, dementia [18-22], Alzheimer disease [23], depressive burden [24], stroke [25,52], hip fracture [26], frailty [8,27-29], and traumatic brain injury [30,31]) and specific insurance or socioeconomic statuses (eg, Medicare [24,32,33,35], Medicaid [34,36], or special social support beneficiaries [37]) were reported. The categorization of older adult populations was not limited to disease categories alone but also included factors related to medical service use and socioeconomic status. It was observed that the diseases affecting the study participants predominantly fell within the *noninfectious disease* category.

#### NHA Definitions and Other Outcomes

NHA was defined in different ways across the studies; 6 studies defined *admission* based on a time period after the receipt of acute care (ie, admission to long-term care at 1 y after first-ever ischemic stroke or intracerebral hemorrhage [25]; admission to a nursing home within 1 y of discharge from hip fracture surgery [26]; NHA at 90 d outside of acute care [38]; 1-y home time, which was defined as the number of days spent outside of a nursing home after stroke [52]; and 90 or 100 d of NHA after a traumatic brain injury experience [30,31]). Further, 1 study [39] used a distinctive outcome measure to define NHA, which was “whether the subject was recommended by healthcare experts to live in residential care.”

Several studies used other outcome measures in addition to NHA (Table 2). All-cause mortality (studies: 16/34, 47%), as well as hospitalization and rehospitalization (studies: 11/34, 32%), was frequently used. In other studies, discharge from home and home-based services or recommendations to live in the community were compared to NHA (studies: 4/34, 12%). Further, of the 34 studies, 4 (12%) used 1 or more functional measures, including quality of life, activities of daily living (ADL), and mental well-being. Other measures were discharge to any health care institutions other than a nursing home (eg, rehabilitation center), other long-term care service use, an emergency department visit, the onset of important chronic conditions, fall incidence, time of death, and health and social service costs.

**Table 2.** Outcome measures used in the selected studies beyond nursing home admission (N=37^a^).

Secondary outcome	Outcome reported, n (%)	
Mortality	16 (43)	
Hospitalization or rehospitalization	11 (30)	
Discharge from home and home-based services or recommendations to live in the community	4 (11)	
Functional measures (quality of life, activities of daily living, and mental well-being)	4 (11)	
Discharge to any health care institutions other than a nursing home (eg, rehabilitation center)	2 (5)	
Other long-term care service use	1 (3)	
Emergency department visit	1 (3)	
Disability	1 (3)	
Onset of 5 important chronic conditions	1 (3)	
Nursing care needs	1 (3)	
Time of death	1 (3)	
Fall incidence	1 (3)	
Health and social service costs	1 (3)	

a More than 1 outcome measure was reported from individual studies. Therefore, N was greater than the number of selected studies.

#### Predictors of NHA

A wide range of predictors was identified across the reviewed studies (Table 3). Some studies used sociodemographic predictors, such as age, sex, residence, marital status, migration status, household income, preferential status in public health care insurance, and living alone. Caregiver factors were measured in 4 studies, including the type of informal care [21,23,39,40]. Health-related factors included cognitive function and dementia (eg, cognitive ability, dementia severity, cognitive dysfunction [41], and presence of dementia [26]), falls [20,36], behavioral symptoms at baseline [23], Parkinson disease [26], physical comorbidities [26], stroke [25], depression [20,26], quality of life measured by the EuroQol EQ-5D-3L [20], and the Cumulative Illness Rating Scale [26]. Medication-related variables included the Medication Appropriateness Index [20], the use of antipsychotics [19], dementia medication [22], and medication adherence [42]. Polypharmacy was used to predict NHA in 2 studies [22,43]. On the other hand, frailty was used in 8 studies. Among these 8 studies, 5 reported the development and validation of prediction models using different frailty index scores [27,28,33,35,44]. Another structured score that was used to predict NHA was the Identification of Seniors at Risk score, which is a standardized, comprehensive geriatric assessment tool administered at admission to a hospital [38]. Social service use predictors included short-stay service use, social care type, care needs, the intensity of formal home support [41], and preinjury home care [26]. Additionally, 1 study included the different patterns of use of home- and community-based social services [37].

**Table 3.** Identified predictors of nursing home admission.

Predictors		Items
Sociodemographic factors		Age, sex, residence, marital status, migration status, household income, preferential status in public health care insurance, and living alone
Caregiver factors		Caregiver distress, caregiving coresidence, type of informal care, and relationship with the main caregiver
**Health status**		
	Frailty	Presence of frailty and structured frailty index
	Geriatric conditions (other than cognitive function and dementia)	Presence of 1 or more geriatric syndromes (fall-related fractures, incontinence, and pressure ulcers) and number of previous falls Combined index of geriatric conditions (falls, difficulty in walking, malnutrition, weight loss, vision, decubitus ulcer, severe issues with bladder control, and absence of fecal control), and standardized comprehensive geriatric assessment results
	Cognitive function and dementia	Cognitive ability, dementia severity, decline in dementia severity, cognitive dysfunction, and presence of dementia
	Other diseases and morbidities	Behavioral symptoms, stroke type, depression, Parkinson disease, experience of traumatic brain injury, disease count, and Cumulative Illness Rating Scale
	Quality of life	EuroQol EQ-5D-3L instrument
Health use and medication		Medication Appropriateness Index, medication adherence, use of antipsychotics, dementia medication, polypharmacy, and quality of primary care experience
Social service use		Use of short-stay services, social care type, care needs, intensity of formal home support, preinjury home care, and different patterns of use of home- and community-based services

Administrative claims data were used in 20 (59%) of the 34 studies to capture the predictors of NHA. Only 3 studies explicitly reported the utilization of EHRs as their data source. Predictors were used to predict risk scores for NHA, but none of the studies reported whether the prediction models were implemented in the real world to influence older adults’ decision-making regarding NHA.

#### Modeling Methods Used to Predict NHA

Of the 34 selected studies, 35% (n=12) reported Cox proportional hazards models for their primary modeling method, while 26% (n=9) reported logistic regression models. Further, 6 (18%) studies used both Cox proportional hazards models and logistic regression models. Other regression models used included the Fine-Grey regression model, the subdistribution hazards model, the generalized linear model, and survival analysis using the competing risk method. (Table 4).

**Table 4.** Modeling methods used in the risk prediction of nursing home admission (N=34).

Modeling methods	Method reported, n (%)	
Cox proportional hazards model	12 (35)	
Logistic regression model	9 (26)	
Cox proportional hazards and logistic regression model	6 (18)	
Other regression models (Fine-Grey regression model, subdistribution hazards model, GLM^a^, and survival analysis using the competing risk method)	5 (15)	
Linear and logistic regression models	1 (3)	
Receiver-operator curve analysis	1 (3)	

a GLM: generalized linear model.

#### Location of Studies

The United States was the country with the highest number of studies (8/34, 24%). In Europe, 9 countries published 12 studies, including studies conducted in Germany (4/34, 12%). Further, of the 34 studies, 4 (12%) were conducted in Asia. Additionally, 1 (3%) study was conducted in Australia, and 1 (3%) study was conducted in more than 1 continent (Table 5).

**Table 5.** Countries and regional distribution of selected studies (N=34).

Regions and countries		Studies, n (%)	
**United States and Canada**			
	United States	8 (24)	
	Canada	4 (12)	
**Europe**			
	Germany	4 (12)	
	United Kingdom	2 (6)	
	Spain	1 (3)	
	Denmark	1 (3)	
	Ireland	1 (3)	
	Finland	1 (3)	
	The Netherlands	1 (3)	
	Belgium	2 (6)	
	Italy	2 (6)	
	Switzerland	1 (3)	
**Asia**			
	South Korea	2 (6)	
	Japan	1 (3)	
	Taiwan	1 (3)	
**Australia**			
	Australia	1 (3)	
**>1 continent**			
	Italy, Spain, Germany, the Netherlands, France, Czech Republic, and Australia	1 (3)	

#### Major Findings of Studies on NHA Prediction

A total of 5 studies included validated frailty indexes as predictors (Table 6). Studies that used a frailty assessment to develop a risk index exhibited similarities in terms of objectives, variables, and outcome measures when compared to studies that reported a distinct, heterogeneous set of predictors. According to the reviewed studies, a frailty index could be adapted for use with EHR data or claims data to predict NHA. According to Clegg et al [44], the primary aim of an electronic frailty index is to identify categories of frailty; therefore, the assessment of utility should be based primarily on the predictive validity of frailty categories for adverse outcomes. Kinosian et al [33] reported that the area under the curve for predicting NHA based on the JEN frailty index was 0.781, although it discriminated less well than an ADL-based model. Segal et al [35] reported that a claims-based frailty indicator significantly predicted NHA in an adjusted model (odds ratio 1.45, 95% CI 1.04-2.01). Clegg et al [44] reported that an EHR-based frailty index identified older people with severe frailty and showed robust predictive validity for outcomes of NHA (hazard ratio 4.76, 95% CI 3.92-5.77).

**Table 6.** Studies that included validated frailty prediction models in NHA^a^ predictions.

Study (author, year)	Prediction model	Data source	Sample size for NHA prediction, n	Area under the ROC^b^ for NHA
Amuah et al [28], 2023	FRM^c^	Clinical administrative data	788,701	0.81
Le Pogam et al [27], 2022	eFS^d^	Clinical administrative data	469	0.71
Kinosian et al [33], 2018	JFI^e^	Administrative claims (Medicare)	12,563	0.78
Segal et al [35], 2017	CFI^f^	Administrative claims (Medicare)	4454	0.75
Clegg et al [44], 2016	eFI^g^	EHR^h^ (primary care setting)	931,541	0.74

a NHA: nursing home admission.

b ROC: receiver-operator curve.

c FRM: frailty risk measure.

d eFS: electronic frailty score.

e JFI: JEN Frailty Index.

f CFI: claims-based frailty indicator.

g eFI: electronic frailty index.

h EHR: electronic health record.

A total of 3 studies reported the usefulness of calculating a risk index or predicting NHA. Kan et al [32] reported that the odds ratio estimates for their geriatric risk index were statistically significantly associated with increased health care utilitzation, including NHA in the first year of study. Pilotto et al [45] reported that a higher Multidimensional Prognostic Index value was associated with NHA and other negative outcomes. Müller et al [20] reported a comparison between claims data–based and trial data–based prognostic models to predict negative health outcomes in older patients with multimorbidity and polypharmacy.

Predictors that were identified to decrease the risk of NHA included the receipt of higher-quality primary care, the receipt of coresident care, integrated medical care and other types of long-term care services, intense formal home support, and short-stay service use (Table S5 in Multimedia Appendix 1).

## Discussion

### Principal Findings

In this study, we reviewed 34 studies that either assessed the association between predictors and outcomes or reported the development and validation of predictors of NHA via observational studies based on EHR or administrative data. These predictors were further categorized into sociodemographic, caregiver support, health status, health use, and social service use factors, consistent with previous studies [9,18-22,24,26,34,39,40,43,46,52,53]. Most studies (23/34, 68%) included multiple outcome variables in addition to NHA. All-cause mortality, hospitalization, and rehospitalization were frequently used as outcome measures. Cox proportional hazards models, logistic regression models, and a combination of both were frequently used to predict NHA. In terms of data sources, administrative claims data and EHRs were jointly used, rather than each source being used alone.

Overall, studies did not fully leverage the data sources of our interest. Administrative claims and EHRs may have strengths, including the abilities to help with recruiting larger sample sizes, incorporate expanded types of predictors or outcome variables, enable short-term prediction, and facilitate easy implementation in practice [54]. For instance, routinely coded EHR data can be used to establish a frailty index, thereby allowing for the classification of subtypes of frailty. These data can be readily integrated into clinical computer systems, automatically populated, and made available without requiring additional resource input [44]. Further, although we found some studies that used large sample sizes comprised of more than 200,000 participants [18,20,28,31,43,44,47], this did not necessarily mean that the predictors or outcome variables were distinctive. In addition, although many studies used administrative claims data, the use of billing information or the construction of cost predictors was rarely observed. Discussion on enhancing the quality of data source utilization was limited. For instance, only 1 study reported the variation in outcomes based on the use of structured EHRs versus nonstructured EHRs [32].

Developing a validated risk prediction model that is expressed as an index or score is one option to effectively leverage electronic data sources. Such a model would serve as a convenient tool for preplacement assessments and help address the challenges posed by big data sources, which often contain excessive information that may be disorganized or lack systematic summarization. As international guidelines recommend the routine identification of frailty to provide evidence-based treatment [44], several studies tried to identify frailty from routinely available electronic health data, which showed fair predictive value. Despite the identified NHA predictors, the development of a predictable index from routine data sources needs further exploration, as the predictive value of the identified factors was limited.

Although many of the reviewed studies included clinical predictors, social determinants of health, such as education, economic hardships, food security, and community factors [55], were used in a limited fashion. The limited use of social determinants of health might be partly due to the complexities of extracting such data from EHRs and the limited capture of these data in insurance claims [56]. Similarly, caregiving, which is an important aspect of social support for patients, was used as the predictor of NHA in a few of the reviewed studies. One study [21] collected information on a caregiver’s formality and qualitative characteristics of caregiving (eg, value of caregiving to and impact of caregiving on care recipients). In another study [40], a comprehensive assessment survey, which included the caregiver distress factor, was conducted and included as a predictor of NHA.

The study findings have implications for the development of prevention strategies in communities. Several studies reported the utilization of home care to delay NHA [8,36-39,41,48,49]. Prediction models for older adults may play a distinctive role in screening the at-risk population or assessing the need for home services in primary care settings. Efficient NHA prediction may involve making the short-term decision of whether a person is suited for living in the community or for institutionalization and applying this approach to a specific population instead of applying it broadly to all community-dwelling older adults. In addition, such complex decision-making may require informed communication among older adults, caregivers, and care providers about resource allocation between community-based care and institutional care. Therefore, NHA prediction in communities should be in alignment with the continuum of care, considering the benefits and costs of institutionalization.

### Comparison to Prior Work

Unlike other recent studies that primarily targeted risk prediction for nursing home residents [57,58], this study examined the preplacement status of individuals, with many of the study samples residing in communities. Our focus was on exploring how the study results could assist in developing prevention strategies at the population health level within these communities.

The main findings of this study are consistent with a previously conducted meta-analysis [9] and cohort study [53], which identified predictors of NHA, specifically ADL dependency, cognitive decline, and prior NHA. Studies on the predictors of readmission to nursing homes also showed similar trends [59]. Nevertheless, in comparison to these prior studies, our study revealed an expansion in the types of predictors reported since 2010, including frailty and geriatric syndromes.

Previous studies on risk prediction via EHR data or administrative claims data discussed various strengths and challenges [48,54,60,61]. EHRs may provide an alternative to the longitudinal cohort studies that are traditionally used to construct risk models [60]. In this regard, clinically available risk prediction models for the early prevention of NHA that are based on EHRs are more appropriate for a clinical context than those based on other types of electronic data sources [44]. However, variations in EHR implementations and data standards across health care systems may limit the practical adoption of EHR-based NHA prediction models [62]. Future research should pay attention to the development and use of predictors of NHA across different electronic data, health systems, and patient populations.

### Strengths and Limitations

To our knowledge, this is the first study to systematically review existing evidence on identifying predictors of NHA based on administrative claims or EHRs. Since NHA is a considerable burden to health care delivery systems, identifying and developing NHA prediction models can enable health systems to both reduce overall health service utilization and improve health care outcomes among older adults. We reported distinctive characteristics of existing predictors and prediction models, including how they leveraged specific data sources. These findings have implications for delaying NHA in communities.

This review has several limitations. First, this review is limited to the literature on EHRs and administrative data concerning the older adult population and NHAs. Therefore, the findings from the literature on EHRs and administrative claims data may not be generalizable to other data sources. Second, this review only included studies that researched potential factors or predictors of NHA in a retrospective manner. Thus, studies that evaluated a specific program for reducing NHAs were excluded in this study. These studies may have included predictors that could have been added to the list of predictors found in this review, although such predictors are often not available in routine EHR data or claims data sources. To mitigate this limitation, we searched and reviewed the list of excluded studies during the multiple stages of the final selection process. Third, we limited this review to studies that used administrative data or EHR data as their primary sources of data to assess predictors of NHA. However, the differences between administrative data, administrative claims data, and EHR data are not always clear in different clinical settings. Future studies may refine data sources beyond EHRs and claims data, although the application of such data sources in population health management efforts should be further evaluated. Finally, our review followed a scoping review guideline and thus may have inadvertently excluded a few relevant studies. A systematic review of relevant studies is recommended to provide a comprehensive list of predictors for NHA among older adults.

### Future Directions

Considering that NHA is not an end point but rather a transitional point [9] in the care continuum, an NHA prediction model can be used in a risk prediction and prevention framework (Figure S1 in Multimedia Appendix 1). In the first phase of preventing NHA, adults may undergo routine screenings while maintaining independence in their communities. If they experience functional decline or reach a certain level of dependency, preventive interventions can be implemented based on the results of prediction models to delay NHA timing in the second phase of prevention. Even after NHA occurs, prevention strategies to reduce the risk of other adverse outcomes during nursing home residency are still important. During this phase of prevention, we believe that NHA risk prediction models can evaluate whether an individual is prepared to return to the community, if relevant. Thus, risk scores calculated by using a frailty index or a comprehensive geriatric assessment index can also be used for newly admitted nursing home residents. In future studies, other factors may be controlled to examine the impact of using NHA prediction scores (Figure S2 in Multimedia Appendix 1).

Of note, some studies focused on continuous care and primary care qualities [8,44,55]. As prevention programs for delaying NHA at earlier stages can be implemented in primary care settings, further studies are warranted on this topic.

### Conclusions

Several studies have investigated the potential predictors of NHA by using EHRs or administrative data. Frequently used predictors included sociodemographic factors, caregiver factors, health-related factors, medications, and medical and social service utilization. The most frequently used models for predicting NHAs were Cox proportional hazards models and logistic regression models. Prediction tools based on EHR data or administrative claims data may assist clinicians, patients, and policy makers in making informed decisions and allocating public health resources.

## Supplementary material

10.2196/42437 Multimedia Appendix 1 Supplementary materials, including those regarding eligibility criteria, search terms, data charting results, predictors of nursing home admission (NHA), an NHA framework, and the utilization of NHA risk scores.

10.2196/42437 Checklist 1 PRISMA-ScR (Preferred Reporting Items for Systematic Reviews and Meta-Analyses extension for Scoping Reviews) checklist.

## Abbreviations

ADL: activities of daily living
EHR: electronic health record
NHA: nursing home admission
PRISMA-ScR: Preferred Reporting Items for Systematic Reviews and Meta-Analyses extension for Scoping Reviews
SNF: skilled nursing facility
