# Correction: Identifying Predictors of Nursing Home Admission by Using Electronic Health Records and Administrative Data: Scoping Review

**DOI:** 10.2196/54952

**Published:** 2023-12-19

**Authors:** Eunkyung Han, Hadi Kharrazi, Leiyu Shi

**Affiliations:** 1 Ho-Young Institute of Community Health, Paju, Republic of Korea; 2 Asia Pacific Center For Hospital Management and Leadership Research, Johns Hopkins Bloomberg School of Public Health, Baltimore, MD, United States; 3 Department of Health Policy and Management, Johns Hopkins School of Public Health, Baltimore, MD, United States; 4 Division of Biomedical Informatics and Data Science, Johns Hopkins School of Medicine, Baltimore, MD, United States

In “Identifying Predictors of Nursing Home Admission by Using Electronic Health Records and Administrative Data: Scoping Review” (JMIR Aging 2023;6:e42437) the authors noted one error.

In the originally published manuscript, some percentage values in Table 2 were listed incorrectly. The corrected version of Table 2 appears as follows:

**Table 2.** Outcome measures used in the selected studies beyond nursing home admission (N=37^a^).

Secondary outcome	Outcome reported, n (%)
Mortality	16 (43)
Hospitalization or rehospitalization	11 (30)
Discharge from home and home-based services or recommendations to live in the community	4 (11)
Functional measures (quality of life, activities of daily living, and mental well-being)	4 (11)
Discharge to any health care institutions other than a nursing home (eg, rehabilitation center)	2 (5)
Other long-term care service use	1 (3)
Emergency department visit	1 (3)
Disability	1 (3)
Onset of 5 important chronic conditions	1 (3)
Nursing care needs	1 (3)
Time of death	1 (3)
Fall incidence	1 (3)
Health and social service costs	1 (3)

a More than 1 outcome measure was reported from individual studies. Therefore, N was greater than the number of selected studies.

The originally published version of Table 2 is included as Multimedia Appendix 1.

The correction will appear in the online version of the paper on the JMIR Publications website on December 19, 2023, together with the publication of this correction notice. Because this was made after submission to PubMed, PubMed Central, and other full-text repositories, the corrected article has also been resubmitted to those repositories.

## Supplementary material

10.2196/54952 Multimedia Appendix 1 Originally published Table 2.

