# Supplementary material for: Identifying Predictors of Nursing Home Admission by Using Electronic Health Records and Administrative Data: Scoping Review
Source: JMIR Aging. 2023 Nov 20;6:e42437. doi: 10.2196/42437 (PMC10686617; doi:10.2196/42437)
Supplement: Multimedia Appendix 1 [file aging-v6-e42437-s001.doc]

**Supplementary materials**

**Table S1.** Eligibility criteria and search terms used in PubMed.

| Eligibility criteria | Search terms |
| --- | --- |
| Population | ("institutionalization"[MeSH Terms] OR "nursing homes"[MeSH Terms] OR "long-term care"[MeSH Terms] OR "institutionalisation"[All Fields] OR "institutionalise"[All Fields] OR "institutionalised"[All Fields] OR "institutionalising"[All Fields] OR "institutionalism"[All Fields] OR "institutionalization"[MeSH Terms] OR "institutionalization"[All Fields] OR "institutionalizations"[All Fields] OR "institutionalize"[All Fields] OR "institutionalized"[All Fields] OR "institutionalizing"[All Fields] OR "nursing home*"[Title/Abstract] OR "long-term care"[MeSH Terms] OR "long term care"[Title/Abstract] OR "LTC"[Title/Abstract] OR "skilled nursing facilities"[MeSH Terms] OR "skilled nursing facilit*"[Title/Abstract] OR "SNF"[Title/Abstract] OR "homes for the aged"[MeSH Terms] OR "homes for the aged"[Title/Abstract] OR ("homes"[Title/Abstract] AND "aged"[Title/Abstract]) OR "residential facilities"[MeSH Terms] OR "subacute care"[MeSH Terms] OR "sub-acute care"[Title/Abstract] OR "post acute care"[Title/Abstract] OR "post-acute care"[Title/Abstract] OR "long-term care facilit*"[Title/Abstract] OR "care home"[Title/Abstract] OR "home time"[Title/Abstract] NOT "hospices"[MeSH Terms]) AND ("admission*"[Title/Abstract] OR "readmission*"[Title/Abstract])  AND ("older adults"[Title/Abstract] OR "aged"[MeSH Terms] OR "aged"[Title/Abstract] OR "elderly"[Title/Abstract] OR "elderlies"[Title/Abstract] OR "medicare"[MeSH Terms])  (6,692 results, 2023/03/31) |
| Concept | "risk"[Title/Abstract] OR "predict*"[Title/Abstract] OR "model*"[Title/Abstract] OR "analytic*"[MeSH Terms] OR "forecast*"[Title/Abstract] OR "simulation"[Title/Abstract] OR "area under curve"[All Fields] OR "AUC"[All Fields] OR "linear model*"[All Fields] OR "logistic model*"[All Fields] OR "proportional hazards model*"[All Fields] OR "ROC"[All Fields] OR "survival analysis"[All Fields] OR "kaplan-meier estimate*"[All Fields] OR "hazard ratio"[All Fields] OR "odds ratio"[All Fields] OR "machine learning" [Title/Abstract] OR "artificial intelligence"[Title/Abstract] OR "dynamic prediction" [All Fields]  (22,901,532 results, 2023/03/31) |
| Context | "electronic health record"[Title/Abstract] OR "electronic medical record"[Title/Abstract] OR "EHR"[Title/Abstract] OR "EHRs"[Title/Abstract] OR "EMR"[Title/Abstract] OR "EMRs"[Title/Abstract] OR "administrative data"[Title/Abstract] OR "claim*"[Title/Abstract]  (161,519 results, 2023/03/31) |
| Timeline | #1 AND #2 AND #3 AND ("2012/01/01"[Date - Publication]: "2023/03/31"[Date - Publication])  (441 results, 2023/03/31) |

**Table S2.** Eligibility criteria and search terms used in CINAHL.

| Eligibility criteria | Search terms |
| --- | --- |
| Population | ( "institutionalization" OR (MH "nursing home*") OR (MH "long-term care") OR "institutionalisation" OR (MH "institutionalization") OR "LTC" OR "skilled nursing facilities" OR "skilled nursing facilit*" OR "SNF" OR "homes for the aged" OR "homes for the aged" OR "(homes" AND "aged") OR "residential facilities" OR "subacute care" OR "sub-acute care" OR "post acute care" OR "post-acute care" OR "long-term care facilit*" OR "care home" OR "home time" NOT "hospices" AND ("admission*" OR "readmission*") ) AND ( "older adults" OR (MH "aged") OR (MH "elderly") OR "elderlies" ) |
| Concept | ("risk" OR "predict*" OR "model*" OR "analytic*" OR "forecast*" OR "simulation" OR "area under curve" OR "AUC" OR "linear model*" OR "logistic model*" OR "proportional hazards model*" OR "ROC" OR "survival analysis" OR "kaplan-meier estimate*" OR "hazard ratio" OR "odds ratio" OR "machine learning" OR "artificial intelligence" OR "dynamic prediction") |
| Context | "AB ( ("electronic health record") OR ("electronic medical record") OR ("EHR") OR ("EMR") OR ("administrative data") OR "claim*" ) |
| Limiters | Published Date: 20120101-20211231; Exclude MEDLINE records; Language: English  Expanders - Apply equivalent subjects  Search modes - Boolean/Phrase |

**Table S3.** Data charting of identified studies for scoping review (N=34).

| **Author(s)** | **Study sample** | **Country** | **Dependent variables** | **Independent variables** | **Data source(s)** | **Statistical Analysis** | **Findings or conclusions** |
| --- | --- | --- | --- | --- | --- | --- | --- |
| Temple J, et al. [39] | 280,000 | Australia | Recommendations to live in the community or residential care | Type of informal care (carer residence & relationship with the study participant) | Administrative data on aged care assessments from the National Aged Care Data Clearing House | Logistic regression models | Co-resident care plays an important protective role in residential care admission. Weaker non-coresidential friend or neighbor carer relationships are associated with recommendations to live in residential care for women. In addition, health conditions and assistance needs play a strong role in assessor recommendations about entry to residential care. |
| Byrne, et al. [34] | 432 | US | Nursing home admission | Demographic characteristics, health and behavioral health conditions, and health services utilization measures | Administrative data from the emergency shelter system in Boston, MA and claims data from the Massachusetts Medicaid program | Cox proportional hazards regression models | Older age, diagnoses indicating alcohol use disorder, greater overall disease burden, and a prior history of nursing home admission were all associated with a higher risk of nursing home admission following shelter entry. |
| Wahlsten, et al. [26] | 53,157 | Denmark | Admission to nursing home within one year of discharge from hip fracture surgery | Age, living alone, dementia, pre-injury home care, Parkinson's disease and depression, other physical comorbidities | A nationwide administrative Dannish registries | Cumulative incidence curves and Cox regression models | One-year risk increased with advancing age. Living alone and dementia were strong risk factors. Other important risk factors were pre-injury home care, Parkinson’s disease and depression. Physical comorbidities i.e. kidney disease, chronic obstructive pulmonary disease, diabetes and cancer did not increase the risk of nursing home admission. |
| Van den Bosch K, et al. [46] | 69,562 | Belgium | First use of home long-term care and residential care; onset of five important chronic conditions and the time of death. | Preferential status (a status that entitles persons to higher reimbursement rates for health care from the public health care insurance system and is conditional on low income) | Administrative panel of a sample representative for all older persons in Belgium | Logistic regression and Cox proportional hazards model | A strong association was found between preferential status and the likelihood of home care use, but for  residential care it is small for men and non-existent for women. Preferential status was significantly related to the chance of getting chronic conditions and to the probability of dying (not for women). For home care use and death, the association with preferential status declines with increasing age from age 65 onwards, such that it is near zero for those aged around 90 and older. |
| Betini RSD, et al. [40] | 94,957 | Canada | Long-Term Care Home (LTCH) admission | Care needs, age, caregiver distress, caregiving co-residence | Ontario Health Networks data | Cox proportional hazards models | Care recipient health care needs and age were the strongest predictors of LTCH admission followed by caregiver distress and caregiving co-residence and relationship. The results emphasize the influence of caregiver distress in LTCH admission and highlight the  impact of caregiving relationship and co-residence on this outcome. Policy and decision makers should consider these findings when developing and evaluating interventions aiming to avoid LTCH admissions. Moreover, caregiving co-residence and relationship should be explored in future studies. |
| Korhonen K, et al. [18] | 248,078 | Finland | Institutional long-term care use | Age, marital status, and household income | Finnish Death Register and national care registers | Repeated-measures logistic regression models with generalized estimating equations (GEE) | The effects of age, marital status and household income on institutional LTC varied across the time before death, and the patterns differed between dementia-related and non-dementia-related deaths. |
| Byers, et al. [24] | 3,646 | US | Nursing home placement | Depressive symptom burden | Medicare claims files | Fine-Gray proportional hazards analysis | In older women, cumulative burden of depressive symptoms over nearly 2 decades is associated with greater risk of transitioning from community-living  to a NH irrespective of recent depression exposure, medical comorbidities, functional impairment, and the competing risk of death. This work supports the need for improving recognition, monitoring, and treatment of depressive symptoms early, which may reduce or delay NH placement. |
| Nerius, et al. [19] | 6,930 | Germany | Long-term care use, nursing home (NH) admission, death | Use of antipsychotic drugs (APDs) | Health claims data of German health insurer | Cox proportional hazards model | APD users generally faced a twofold increased risk of LTC relative to nonusers. The risk of moving into a NH was generally increased by about 50% among APD users relative to nonusers. Risk of death was significantly higher for haloperidol-, melperone-, and risperidone- but not for quetiapine users. APDs appeared to accelerate adverse health outcomes in German dementia patients. Differentiating between the effect of antipsychotic drug use among dementia patients residing in private households and in NHs, we found that excess mortality for haloperidol and melperone users was higher in private settings. |
| Müller, et al. [20] | 592, 998 | Germany | Health related Quality of Life (HRQoL), a combined outcome of falls, hospitalization,  Institutionalization, nursing care needs | Age, sex, disease count, medication-related variables and health service utilisation. Cumulative Illness Rating Scale, depression, Medication Appropriateness Index (MAI), lifestyle, functional status and HRQoL | Two independent data sets, one  comprising health insurance claims data and the other data from the PRIoritising MUltimedication in  Multimorbidity (PRIMUM) cluster randomised controlled  trial | Mixed (linear and logistic) regression models | This study provides prognostic models to identify older general practice patients with multimorbidity and polypharmacy at high risk of deterioration in HRQoL, hospitalization, falls/fall-related injuries, institutionalization, and a need of nursing care. Outcome components, such as previous falls, hospital stays, reduced HRQoL and depression, were important predictors of these negative health outcomes in our models. A decline in HRQoL, a previous hospitalization and a previous falls/fall-related injury can therefore be seen as a warning parameter that may help general practitioners in recognizing older patients with multimorbidity and polypharmacy at high risk of adverse health outcomes. |
| Runte R. [21] | 652 | Germany | Institutionalization, mortality | Gender, service type, care level, caregiver factors | A survey linked with administrative data | Cumulative incidence function and Sub-distribution hazard model | The regression models show that the risk of institutionalization is higher in women than in men and when cared for by a care service in comparison to an informal caregiver. Inhibiting factors are Care Level and positive evaluation of caregiving by caregivers. Stratified analysis by sex revealed that the risk of institutionalization in men is influenced by their relationship to their caregiver, in women by duration of care at baseline. (fit, mild, moderate, and severe) |
| Joling, et al. [22] | 9230 (cases) and 24,624 (matched controls) | Netherlands | Institutionalization, death | Age, care type, gender, residence, migration status, frailty, polypharmacy, dementia medication | Family physicians' electronic records linked with national administrative databases | Multivariable logistic and Cox proportional hazard model | Older age and receiving home care were the strongest predictors of shorter time until institutionalization and death in people with dementia. Gender, cohabitation, migration status, frailty, polypharmacy, and dementia medication were other significant factors. |
| Park D, et al. [23] | 2,470 | South Korea | Institutionalization to long-term care facility | Sociodemographic factors, cognitive ability, behavioral symptoms at baseline, comorbidities, alcohol drinking and smoking, medication, relationship with the main caregiver, APOE genotype | The public Long-Term-Care Insurance (LTCI) program data of South Korea | Cox proportional hazards models | Lower cognitive ability, higher dementia severity, more-severe behavioral symptoms at baseline, more-rapid decline in dementia severity, and more-frequent use of antipsychotics are independent predictors of earlier institutionalization. |
| Joundi RA, et al. [25] | 163,574 | Canada | Discharge home and to rehabilitation and admission to long-term care at 1 year after first-ever ischemic stroke or ICH | Age, sex, comorbidity, rural residence, stroke severity, ICU admission, tracheostomy, and other patient traits | The Canadian  Institutes for Health Information (CIHI) Discharge Abstract Database | Cox proportional hazard and logistic regression models | Older age, female sex, greater estimated stroke severity,  Charlson score ≥2, feeding tube placement, and tracheostomy were associated with a higher hazard of long-term care admission for both stroke types |
| Kan, et al. [32] | 16,705 | US | Hospitalizations, ed visits, and nursing home visits | Geriatric risk index | EHR and administrative claims for Medicare Advantage program | Logistic regression | The prevalence of geriatric risk factors increased after adding unstructured EHR data to structured EHRs, compared with those derived from structured EHRs alone and claims alone. On the basis of claims, structured EHRs, and structured and unstructured EHRs combined, |
| Kinosian, et al. [33] | 12,563 | US | Long-term institutionalization risk and activities of daily living (ADL) impairments | The JEN Frailty Index: The 13 JFI domains are: minor ambulatory  limitations, severe ambulatory limitations, chronic mental illness, chronic developmental disability, dementia, sensory disorders, self-care impairment, syncope, cancer,  chronic medical disease, pneumonia, renal disorders, and other systemic disorders. The JFI score is the unweighted sum of the condition domains triggered. | 2004 U.S. National Long-Term Care Survey data were linked to Medicare, Minimum Data Set, Veterans Health Administration files and vital statistics | Logistic regression models | The JEN Frailty Index with demographic covariates is a valid claims-based measure of concurrent activities-of-daily-living impairments and future long-term institutionalization risk in older populations lacking functional information. |
| Segal JB, et al. [35] | 4,454 | US | Common aging-related outcomes including nursing home admission, hospitalization, disability, death, and time to death. | The Claims-based Frailty Index (CFI) consisted of variables such as impaired mobility, depression, congestive heart failure, Parkinson’s disease, white race, Charlson’s comorbidity index, etc. | The CHS cohort data linked to participants Medicare claims | Penalized logistic regression models | Claims data alone can be used to classify individuals as frail and non-frail. The CFI might be used in research with large datasets for confounding adjustment or risk prediction. The indicator might also be used for emergency preparedness for identification of regions enriched with frail individuals. |
| Spoelstra, et al. [36] | 6,515 | US | Nursing home placement (NHP) | Prior NHP, falls rate | The Minimum Data Set-Home Care linked with Medicaid claim files | Generalized linear regression models | An adverse event such as a fall and prior NHP is a strong predictor of future NHP and should be taken into consideration while developing care plans for community-dwelling older adults. |
| Wang, et al. [37] | 71,260 | Taiwan | Nursing home admission | The different patterns of use of home- and community-based services | Taiwan's first National 10-Year Long-Term Care Plan database and from National Health Insurance Claim Data | Hierarchical multinomial logistic regression models | The control group had higher risk of admission to a nursing home compared to groups which used integrated medical care and other types of long-term care services into adult day care. |
| Edmans, et al. [38] | 667 | UK | Clinical outcome at 90 days (where an adverse outcome was any of death, institutionalization, hospital readmission, increased dependency in activities of daily living, reduced mental well-being or reduced quality of life (reduction in the EuroQol-5D) and high health and social services costs over 90 days | The Identification of Seniors At Risk (ISAR) score | Routine electronic service records | Receiver-Operator Curve analysis model | Tools are required to identify high-risk older people in acute emergency settings so that appropriate services can be directed towards them. The ISAR tool was poor at predicting adverse outcomes and fair for health and social care costs. The ISAR in older people discharged from acute medical units is unsuitable as a sole tool in clinical decision-making. |
| Aspell N, et al. [41] | 1,597 | Ireland | Admission to long-term care (nursing home care/institution), mortality | Sociodemographic data, physical dependency, medications, cognitive status, intensity of formal home support | Data from the CSAR (The Common Summary Assessment Report) forms: routinely collected  administrative data | Logistic regression models | Cognitive dysfunction and intensity of formal home support were associated with transition to LTC, while physical dependency and advanced age were associated with mortality. Investment in personalised, cognitive‐specific, services and supports are necessary to keep people with dementia and related cognitive impairments living at home for longer. |
| Franchi, et al. [43] | 1,800,257 | Italy | One-year hospitalization, institutionalization, and mortality rates | Incident chronic polypharmacy | The administrative database of the Lombardy region (Northern Italy). | Logistic regression and Cox proportional hazard models | Chronic polypharmacy was significantly associated with the outcomes in multivariable analyses: hospitalization, institutionalization, and death. There was no consistent effect modification by index year or sex, whereas chronic polypharmacy was no longer a risk factor for adverse outcomes among those older than 85 years. |
| Cegri, et al. [48] | 616 | Spain | Inclusion in a nursing home or home care program, mortality | The Comprehensive Geriatric Assessment (CGA) instrument and frailty | Electronic healthcare records, and the Central Registry of Catalonia for mortality | Fine-Grey regression models | Prognostic models based on comprehensive geriatric assessments can predict the need for the commencement of home care and nursing home admission in community-dwelling older adults |
| Clegg A, et al. [44] | 931,541 | UK | Mortality, hospitalization, and nursing home admission (at 1-, 3- and 5- years) | Electronic frailty index (eFI) categories (fit, mild, moderate, and severe frailty) | Primary care EHR data contained in the ResearchOne and The Health Improvement Network (THIN) databases | Cox regression models | The eFI uses routine data to identify older people with mild, moderate and severe frailty, with robust predictive validity for outcomes of mortality, hospitalization and nursing home admission. |
| Pilotto, et al. [45] | 1,140 | Italy, Spain, Germany, The Netherlands, France, Czech Republic, Australia | Mortality, institutionalization, rehospitalization, and use of home care services | A standardized comprehensive geriatric assessment (the CGA-based Multidimensional Prognostic Index) at admission to hospital | Hospital administrative information | Logistic regression models | Higher MPI values are associated with higher mortality and other negative outcomes. Multidimensional assessment of older people admitted to hospital may facilitate appropriate clinical and post-discharge management. |
| Moriyama, et al. [49] | 2,454 | Japan | Residential care admission | Use of short-stay services, service type, sociodemographic factors | Japan Long-Term Care Insurance claims data from Ibaraki Prefecture | Cox proportional hazard models | Use of short-stay services was positively correlated to delay of residential care admission compared to non-use in the low-care need group. In the high-care need group, however, use of short-stay services was somewhat correlated with earlier admission. |
| Kim, et al. [47] | 5,058,720 | Korea | Mortality and institutionalization | Sociodemographic factors, presence of geriatric syndrome, medical history including Charlson’s Comorbidity Index(CCI) and polypharmacy | South Korean National Health Insurance Service claims data | Cox proportional hazards model | Delirium, fall-related fractures, incontinence, and pressure ulcers were associated with increased risks of institutionalization and mortality. The magnitude of these risks increased with increasing numbers of coexisting geriatric syndromes. |
| Le Pogam, et al.[27] | 469 | Switzerland | one-year adverse health outcomes including NHA | Sociodemographic factors at Frailty Phenotype assessment, time  since last hospital discharge, electronic Frailty Score) | claims data of 1) inpatient discharge data from Lausanne University Hospital; 2) 2016 Swiss nationwide hospital discharge data | logistic regression model | Among 34 models tested, the best-subsets logistic regression model with four predictors (age and sex at FP assessment, time since last hospital discharge, eFS) performed best in predicting the dichotomised FP and one-year adverse health outcomes. |
| Seibert, et al. [8] | 18,713 | Germany | time to NHA after initial onset of care-dependency | primary care quality | statutory health insurance claims data (2007–2016) | Multivariable Cox proportional hazard models | Adjusted analyses show consistent  associations of the quality of diabetes care with the duration of remaining in one’s own home regardless of the pres‑  ence of dementia. The quality of primary care provided to care-dependent multimorbid PWD and POWD, influences  the time individuals spend living in their own homes after onset of care-dependency before a NHA. |
| Franchi, et al. [42] | 122,655 | Italy | One-year mortality, nursing home, emergency department (ED), and hospital admission rates | medication adherence by calculating the daily polypharmacy possession ratio (DPPR). | the Lombardy  Region (northern Italy) administrative database from 2016 to 2018 | regression model using Odds Ratio, Cox survival regression model | A higher DPPR was associated with clinical outcomes—in particular, improved survival and lower incidence in nursing home admisꠓsions. Adherence to the most common chronic drugs co-prescribed to the older population was high. Better multiple medication adherence was associated with better clinical outcomes |
| Amuah [28] | 788,701 | Canada | frailty-related adverse outcomes including death, high users of hospital beds, and NHA | Canadian Institute for Health Information (CIHI) Hospital Frailty Risk Measure (HFRM) | CIHI administrative data 2018 to 2019 | Cox survival regression model | The CIHI HFRM showed satisfactory predictive validity and reasonable goodness-of-fit. The CIHI HFRM is a valid tool showing good discriminatory power for several adverse outcomes. |
| Berete, et al. [50] | 1,930 | Belgium | NHA | Socioeconomic factors, use of home care services, history of falls, suffering from urinary incontinence, depression or Alzheimer's disease | health insurance data (2012 tot 2018) | Survival analysis using competing risk method | After multivariable adjustment, higher age, low educational attainment, living alone and use of home care services were significantly associated with a higher risk of NHA. A number of need factors (e.g., history of falls, suffering from urinary incontinence, depression or Alzheimer's disease) were also significantly associated with a higher risk of NHA. |
| Hendin, et al. [29] |  | Canada | mortality, long-term care admission within 1 year | frailty | administrative data (2010-2016) in Ontario | logistic regression | Frailty prior to ICU admission among patients who were eligible for RAI-HC assessment was associated with higher mortality and fewer days spent at home following admission. Frail patients had markedly higher rates of long-term care admission and increased costs per life saved following critical illness. |
| Pappadis, et al. [30] | 26,985 | US | 90-day community residence and to nursing home or SNF | Socioeconomic factors, traumatic brain injury (TBI) experience | Texas Medicare claims data of patients older than 65 years hospitalized for a TBI from January 1, 2014, through December 31, 2017 | logistic regression and Cox survival regression | Patients aged 75+, prior NH residence, dual eligibility, prior TBI diagnosis, and moderate-to-severe injury severity were associated with decreased likelihood of 90-day community residence. |
| Bailey, et al. [31] | 207,355 | US | The NH entry was operationalized as the first NH admission that resulted in a stay ≥100 days. | TBI experience | a 5% sample of Medicare beneficiaries | cause-specific Cox proportional hazards models | In weighted models, beneficiaries with TBI entered NHs at higher rates relative to the non-TBI trauma (HR 1.15; 95% CI 1.10, 1.20) and uninjured (HR 1.67; 95% CI 1.60, 1.74) groups. Future research should focus on interventions to retain older adult TBI survivors within the community. |

**Table S4.** Data charting of identified studies for scoping review (continued).

| **Author(s)** | **Strategies of leveraging administrative claims data or electronic health records (EHRs)** | **Risk score or index** | **Implications for prevention stages** | **Nursing home context in the study (payer/insurer)** |
| --- | --- | --- | --- | --- |
| Temple J, et al. [39] | -Administrative claims data provided more than 280,000 subjects information. | N | Selection of home or NH service recipients (when applying for publicly funded long-term care service) | Public Insurance (Australia) |
| Byrne, et al. [34] | -Administrative data was linked to claims data.  -Claims data were utilized to include ICD codes and NHA. | N | Screening or early prevention (among homeless) | Public Insurance (US, Medicaid) |
| Wahlsten, et al. [26] | -A nationwide administrative data was utilized. | N | Screening or early prevention (among older patients with first time hip fracture) | Public Insurance (Denmark) |
| Van den Bosch K, et al. [46] | -A nationwide administrative data was utilized. | N | Screening or early prevention (among those with lower socio-economic status) | Public Insurance (Belgium) |
| Korhonen K, et al. [18] | -Administrative claims data provided more than 200,000 subjects information.  -Utilized time-varying predictors by using longitudinal data | N | Screening or early prevention (among older patients with dementia) | Public Insurance (Finland) |
| Byers, et al. [24] | -A cohort study data was linked to claims data  -Claims data were utilized to identify NHA | N | Screening or early prevention (among older patients with depression) | Public Insurance (US, Medicare) |
| Nerius, et al. [19] | -Administrative claims data contained ICD codes, medical treatments, NH use and billing information | N | Screening or early prevention (among older patients with dementia) | Public Insurance (Germany) |
| Müller, et al. [20] | -Randomized controlled trial data was linked with claims data.  -Administrative claims data provided more than 590,000 subjects information.  -Authors cite that claims data contained a large number of cases, enabling the prediction model to include many predictors without convergence issues. | N | Screening or early prevention (older patients with multimorbidity and polypharmacy) | Public Insurance (Germany) |
| Runte R. [21] | -A survey data was linked with administrative data. | N | Screening or early prevention (among older patients with dementia) | Public Insurance (Germany) |
| Joling, et al. [22] | -EHR was linked with administrative data.  -In addition to collecting medical information, a frailty index was constructed from EHR.  -Administrative data was utilized to include NHA information. | N | Screening or early prevention (among older patients with dementia) | Public Insurance  (The Netherlands) |
| Park D, et al. [23] | -A cohort study data was linked with administrative data.  -Administrative data was utilized to include NHA information. | N | Screening or early prevention (among older patients with dementia) | Public Insurance (South Korea) |
| Joundi RA, et al. [25] | -Several national administrative databases were linked and utilized, including claims data on NHA information.  -Administrative claims data provided more than 160,000 subjects information. | N | Screening or early prevention (among patients who experienced stroke) | NA (Canada) |
| Kan, et al. [32] | -EHR and administrative claims were linked.  -The outcome of risk index varied according to the type of electronic data: structured/unstructured/combined or alone | Y (geriatric risk score) | Screening or early prevention | Public Insurance (US, Medicare) |
| Kinosian, et al. [33] | -A survey data were linked to administrative claims data. | Y (frailty index) | Screening or early prevention(frailty) | Public Insurance (US, Medicare) |
| Segal JB, et al. [35] | -A cohort study data was linked to administrative claims data. | Y (frailty index) | Screening or early prevention (frailty) | Public Insurance (US, Medicare) |
| Spoelstra, et al. [36] | -The Minimum Data Set was linked with administrative claims data. | N | Selection of home or NH service recipients (when applying for publicly funded long-term care service) | Public Insurance (US, Medicare) |
| Wang, et al. [37] | -A national study database was linked to administrative claims data | N | Selection of home or NH service recipients (when applying for publicly funded long-term care service) | Public Insurance (Taiwan) |
| Edmans, et al. [38] | -Routine electronic service records were utilized including information on frailty and malnutrition | Y (ISAR score) | Selection of home or NH service recipients (when applying for publicly funded long-term care service) | Public Insurance (UK) |
| Aspell N, et al. [41] | -A routinely collected administrative data was used. | N | Selection of home or NH service recipients (when applying for publicly funded long-term care service) | Public Insurance (Ireland) |
| Franchi, et al. [43] | -Administrative claims data provided more than 1,800,000 subjects information.  -Administrative claims data included NH utilization. | N | Screening or early prevention (among older patients having polypharmacy) | Not specified (Italy) |
| Cegri, et al. [48] | -EHR and registry were linked and used. | Y | Selection of home or NH service recipients (when applying for publicly funded long-term care service) | Not specified (Spain) |
| Clegg A, et al. [44] | -EHR based frailty index was developed. | Y (frailty index) | Screening or early prevention(frailty) | Public Insurance (UK) |
| Pilotto, et al. [45] | -Hospital administrative information included NH or home service utilization. | Y (Multidimensional Prognostic Index) | Screening or early prevention | Insurance policy mixed (Europe and Australia) |
| Moriyama, et al. [49] | -Administrative Long-Term Care Insurance (LTCI) claims data provided information of NHA | N | Selection of home or NH service recipients (when applying for publicly funded long-term care service) | Public Insurance (Japan) |
| Kim, et al. [47] | -Administrative Claims data provided more than 5,000,000 subjects information.  -LTCI claims data provided information of NHA. | N | Screening or early prevention (geriatric syndrome) | Public Insurance (South Korea) |
| Le Pogam, et al.[27] | -A number of claims-based frailty scores were compared to the newly developed frailty score. | Y (electronic frailty score) | Screening or early prevention(frailty) | NA (Switzerland) |
| Seibert, et al. [8] | -Administrative claims data comprised of the entire individual health history, including NHA information. | N | Selection of home or NH service recipients (when applying for publicly funded long-term care service) | Public Insurance (Germany) |
| Franchi, et al. [42] | -Administrative data provided more than 1,800,000 subjects information.  -Administrative reimbursement data provided health and NHA information. | N | Screening or early prevention (among older patients having polypharmacy) | Not specified (Italy) |
| Amuah [28] | -Administrative data provided more than 780,000 subjects information.  -Administrative data provided health and NHA information. | Y (hospital frailty measure) | Screening or early prevention(frailty) | Not specified (Canada) |
| Berete, et al. [50] | -A survey data was linked to the health insurance claims data. | N | Screening or early prevention | Public Insurance (Belgium) |
| Hendin, et al. [29] | -A cohort study data was linked to administrative data. | N | Screening or early prevention | Not specified (Canada) |
| Pappadis, et al. [30] | -Administrative claims data was utilized and provided health and NH information | N | Screening or early prevention | Public Insurance (US, Medicare) |
| Bailey, et al. [31] | -Administrative claims data provided more than 200,000 subjects information. | N | Screening or early prevention (among patient experienced traumatic brain injury) | Public Insurance (US, Medicare) |

**Table S5.** Predictors of increased or decreased risk of nursing home admission.

| **Study theme** | **Findings** |
| --- | --- |
| Predictors of increased risk of nursing home admission | higher eFI (frailty) indicator score, higher frailty index score, higher JEN frailty index score, higher geriatric risk index, older age, diagnoses indicating alcohol use disorder, greater overall disease burden, and a prior history of nursing home admission, higher MPI, previous falls/fall-related injuries, previous hospitalizations, number of involved physicians and disease count, living alone and cognitive impairment, lower cognitive ability, higher dementia severity, more-severe behavioral symptoms at baseline, more-rapid decline in dementia severity, more-frequent use of antipsychotics, incident chronic polypharmacy, fall, and prior nursing home placement, lower Activities of Daily Living score, lower Instrumental Activities of Daily Living score, higher social risk score |
| Predictors of decreased risk of nursing home admission | receipt of co-resident care, integrated medical care and other types of long-term care services, intense formal home support, and short-stay service use |


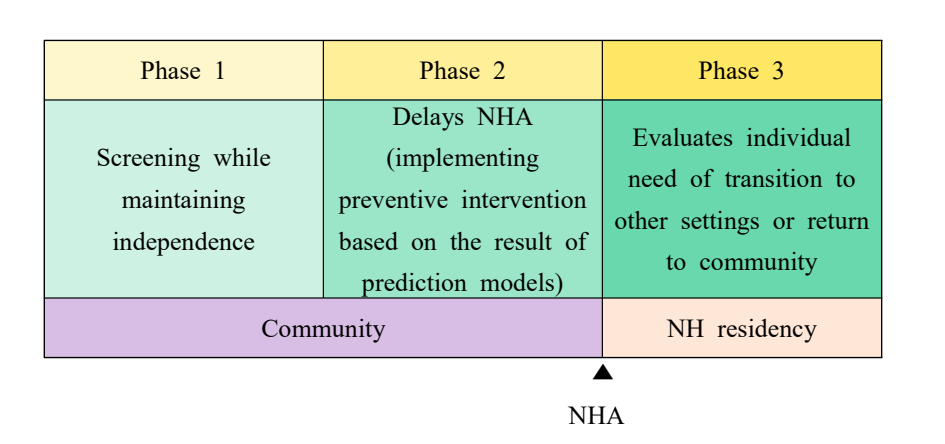


**Figure S1.** A framework of the role of NHA risk prediction models on prevention strategies.


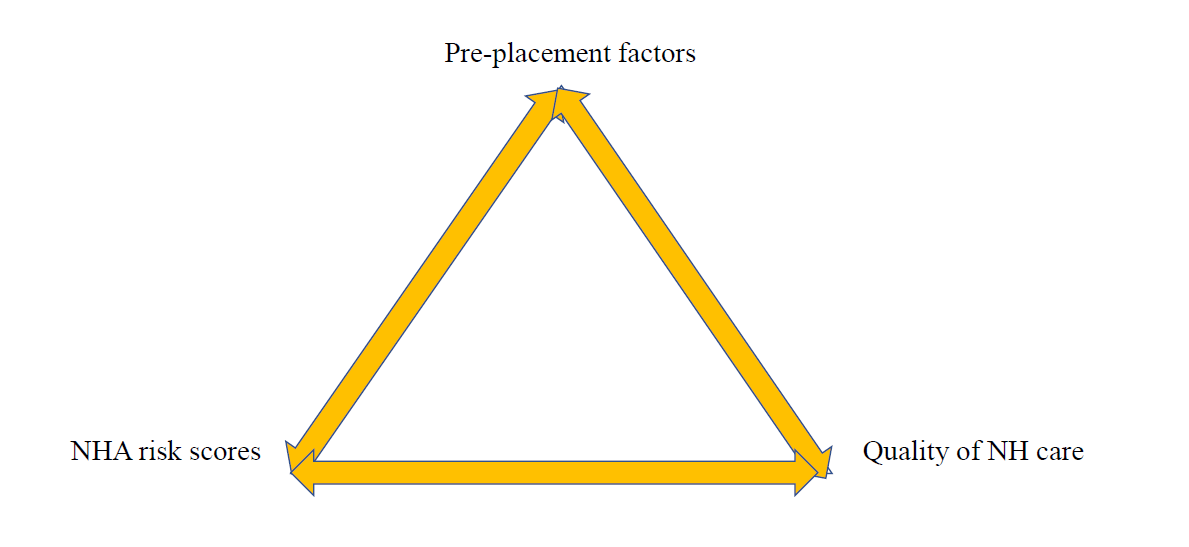


**Figure S2.** Utilization of NHA risk scores for population at risk.
