# Supplementary figures and images for: Correction: Identifying Predictors of Nursing Home Admission by Using Electronic Health Records and Administrative Data: Scoping Review
Source: JMIR Aging. 2023 Dec 19;6:e54952. doi: 10.2196/54952 (PMC10767485; doi:10.2196/54952)

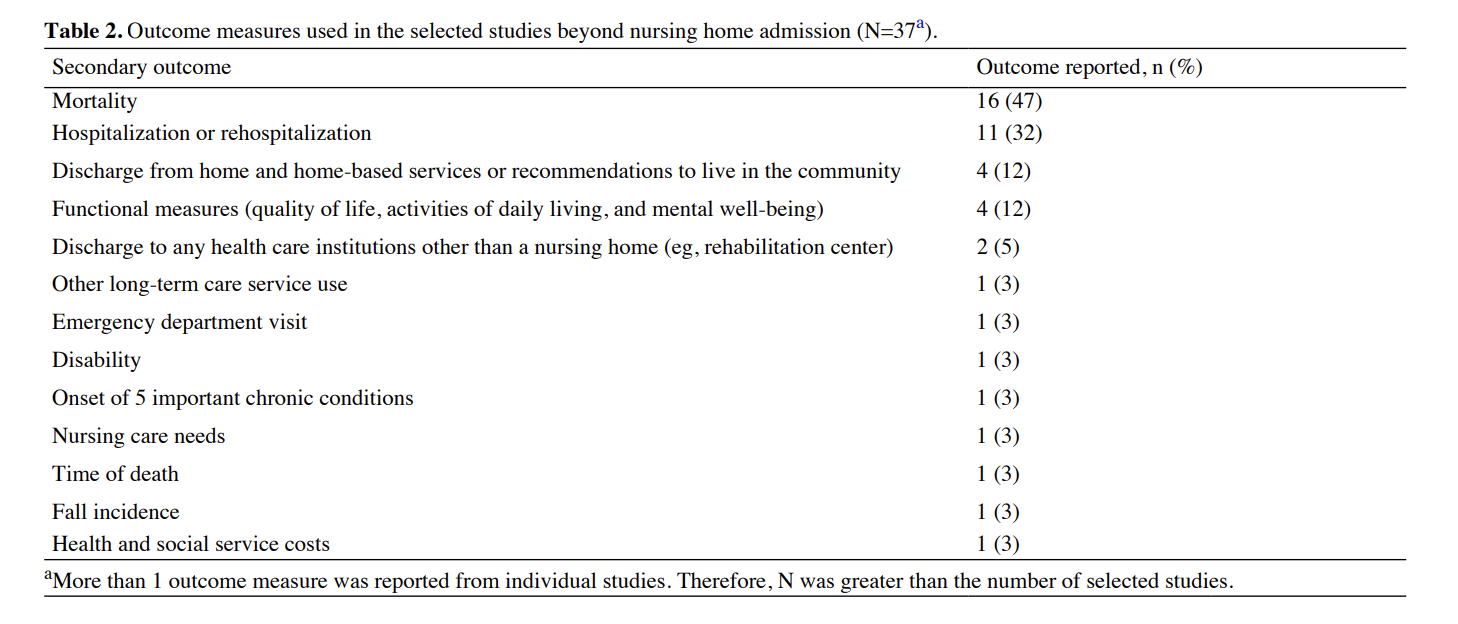

Supplement: Multimedia Appendix 1 [file aging-v6-e54952-s001.docx]
